# Relative Humidity Shifts During Evaporation Enhance the Donor Differentiation Potential of Serum and Plasma Dried Droplet Patterns

**DOI:** 10.3390/molecules31173034

**Published:** 2026-08-28

**Authors:** Maria Olga Kokornaczyk, Dominik Leo Polossek, Zeno Stanga, Mario Castelán, Carlos Acuña, Stephan Baumgartner

**Affiliations:** 1 Society for Cancer Research, 4144 Arlesheim, Switzerland; stephan.baumgartner@unibe.ch; 2 Institute for Complementary and Integrative Medicine, University of Bern, 3010 Bern, Switzerland; 3 Faculty of Philosophy and Natural Sciences, University of Basel, 4001 Basel, Switzerland; 4 Division of Diabetes, Endocrinology, Nutritional Medicine and Metabolism, Inselspital, Bern University Hospital, University of Bern, 3010 Bern, Switzerland; zeno.stanga@insel.ch; 5 Robotics and Advanced Manufacturing, Center for Research and Advanced Studies of the National Polytechnic Institute (CINVESTAV), Ramos Arizpe 25900, Mexico; mario.castelan@cinvestav.edu.mx; 6 EGADE Business School, Tecnologico de Monterrey, 2501 Eugenio Garza Sada Sur, Monterrey 64700, Mexico; carlos.acunaocampo@tec.mx

**Keywords:** droplet evaporation, relative humidity, pattern formation, deep learning, diagnostic pattern test

## Abstract

The droplet evaporation method (DEM) is an analytical approach in which substances dissolved or suspended in a liquid self-assemble into characteristic patterns upon evaporation. Applied to blood and blood-derived fluids, DEM has shown potential for diagnostics. In a previous study, we identified optimal temperature and relative humidity (rH) conditions for generating two main pattern features in dried serum and plasma droplets: crystalline structures (favored at high rH) and peripheral cracks (favored at low rH). The aim of the present study was to investigate whether sequential exposure to two humidity conditions, first 45% rH and then 15% rH, during the evaporation phase of a single experiment could combine both structural features and thereby enhance donor-specific pattern characteristics. Droplets of serum and plasma from eight healthy donors were evaporated under three conditions: constant 45% rH, constant 15% rH, and a changing condition (45% rH for 2 h, followed by 15% rH until completely dry). Evaporation temperatures were 24.5 °C for serum and 30.5 °C for plasma. Computerized texture (GLCM) and fractal (LCFD, MFD, BCFD, lacunarity) image analysis was used to correlate image features with serum and plasma parameters and contents. The donor differentiation was performed by a convolutional neural network (CNN) trained on the droplet pattern images. The changing-humidity condition produced patterns combining the central crystalline structures characteristic of the high-rH condition with the peripheral cracks characteristic of the low-rH condition and yielded the highest CNN-based donor classification accuracy for both fluids, 84.4% for serum, 83.3% for plasma, compared with 66.2–80.8% under the stable conditions. Selected image-evaluation parameters further correlated with the blood derivate contents and parameters including glucose, total protein, triglycerides, homocysteine, urea and the age of the donor. These results suggest that sequential humidity exposure substantially enriches the structural information content of DEM patterns and the amount of donor-specific information that can be extracted from them, with potential implications for diagnostic applications.

## 1. Introduction

When a droplet of a complex fluid dries on a solid substrate, solutes and suspended particles undergo self-organized redistribution, ultimately forming characteristic patterns in the residue [1,2]. The resulting structures depend on a complex interplay of internal factors (fluid composition, viscosity, solute concentration) and external factors (temperature, relative humidity, substrate properties, airflow, and UV radiation) [3,4,5,6]. Because external factors can be controlled, the droplet evaporation method (DEM) enables the extraction of pattern information that reflects the qualitative characteristics of the analyzed fluid.

DEM is applied across a wide range of fields including microelectronics, coating technologies, inkjet printing, DNA mapping, drug screening, bioassay development, synthesis-based analytics, and diagnostics [7,8,9,10,11]. Within the diagnostic domain, patterns formed from desiccated biological fluids, notably blood, serum, plasma, saliva, and tears, have attracted particular interest, as disease states may systematically alter the fluid composition and thus the resulting pattern morphology [12]. Pattern evaluation has progressed from purely visual to computerized image analysis approaches incorporating parameters such as texture (entropy) and fractal geometry (fractal dimension, lacunarity), and more recently towards machine and deep learning [13,14,15,16].

Dried serum and plasma droplets typically exhibit two principal structural features: crystalline structures in the inner zone of the residue and peripheral cracks at the outer rim. A previous systematic study investigated the influence of temperature (18.5–42.5 °C) and relative humidity (15–60% rH) on pattern formation in dried serum and plasma droplets from four donors [2]. That study demonstrated that relative humidity is the key determinant of which structural type predominates: high rH promotes crystalline structures, while low rH favors crack formation. Optimal temperatures were identified as 24.5 °C for serum and 30.5 °C for plasma. These findings established a foundation for the rational optimization of DEM conditions in the context of blood-derived fluid analysis.

A limitation of single-condition DEM experiments is that any given constant humidity selectively enriches one structural type at the expense of the other. Since both crystalline structures and cracks carry potentially independent and complementary information about the sample, an evaporation protocol that combines both conditions sequentially could, in principle, yield patterns with richer information content. Whether such a changing-humidity protocol would indeed produce patterns simultaneously expressing both structural features, and whether this would enhance inter-donor discriminability, has not yet been investigated.

The aim of the present study was therefore to investigate the effect of a sequential two-stage humidity protocol (45% rH followed by 15% rH) on pattern formation in dried serum and plasma droplets, compared with constant high-rH and constant low-rH conditions. We further assessed whether the changing-humidity condition enhances the amount of donor-specific information contained in the resulting patterns, using a convolutional neural network (CNN) trained to recognize donor identity. Computerized texture and fractal image analysis was used to explore correlations between image features and routine blood parameters.

## 2. Results

### 2.1. Visual Evaluation of Droplet Patterns

**Figure 1:**
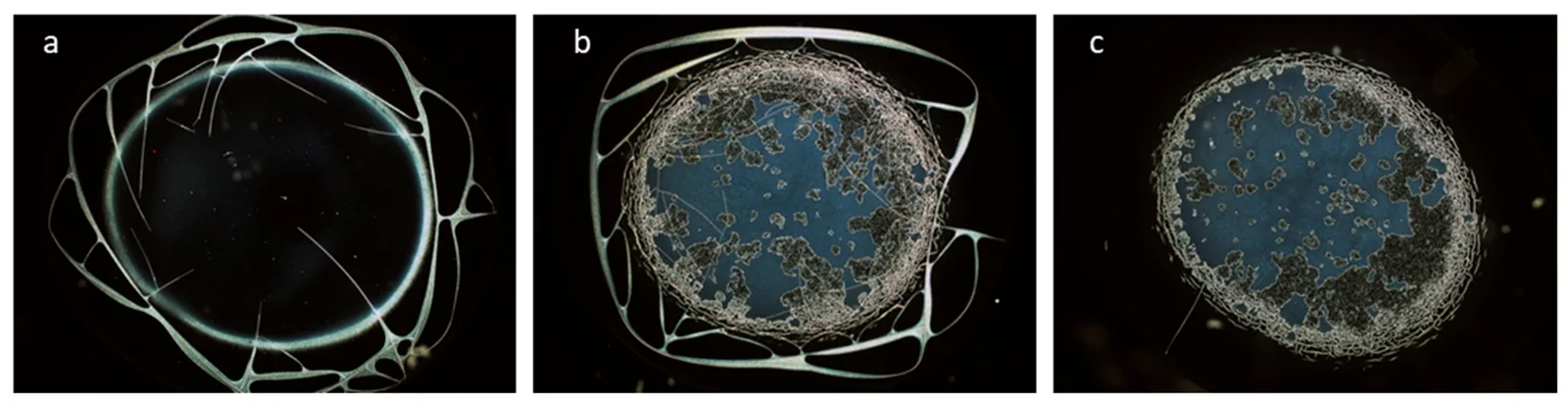
Examples of patterns from serum droplets evaporated under: (**a**) constant 15% rH, (**b**) a relative humidity shift from 45% to 15% rH, and (**c**) constant 45% rH. Notably, the pattern obtained under the shift condition (**b**) combines both kinds of features present in (**a**,**c**).

**Figure 2:**
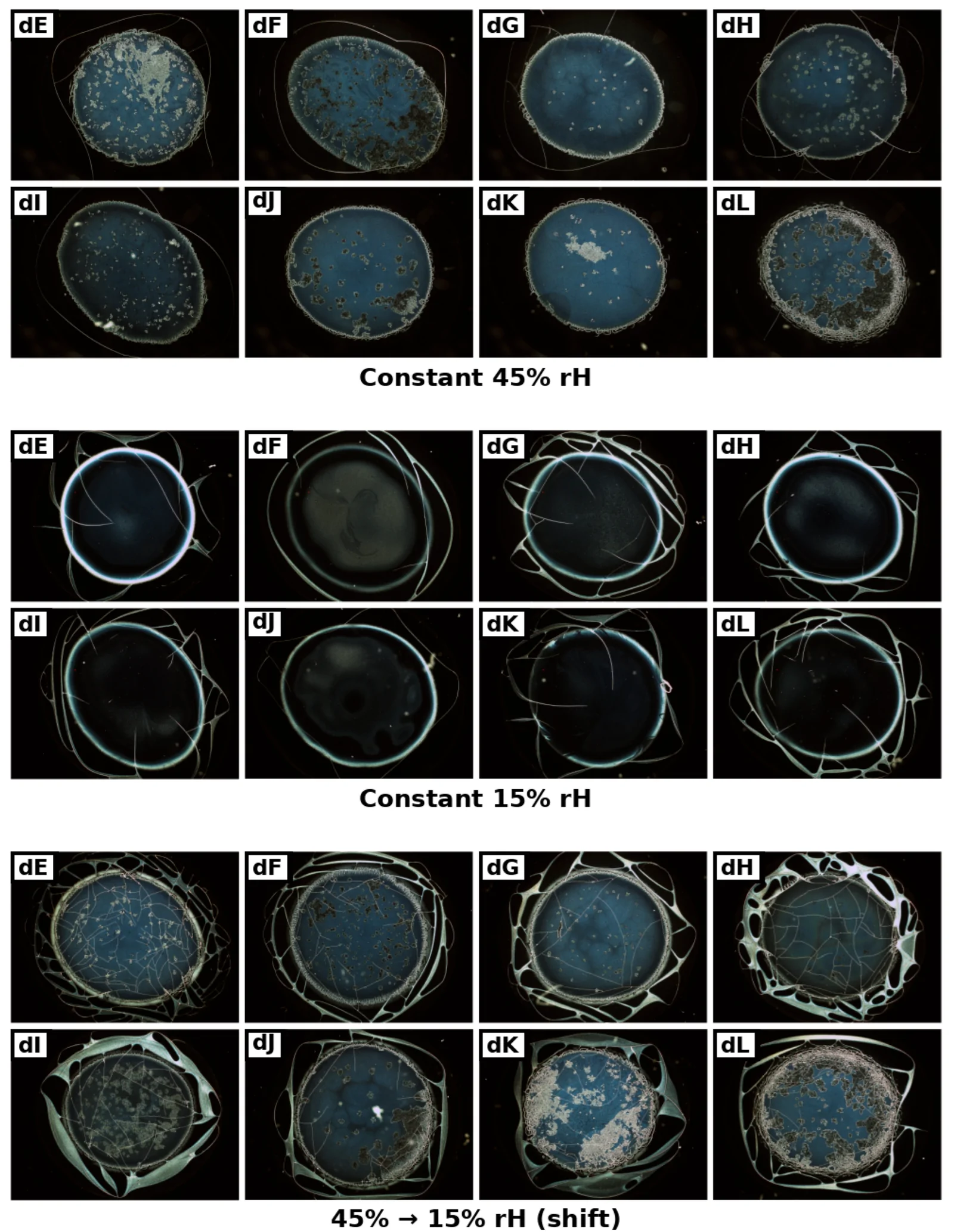
Representative dark-field images of dried serum droplets from all eight donors (dE–dL), shown for each of the three evaporation conditions (**top** to **bottom**: constant 45% rH, constant 15% rH, and the 45% → 15% rH shift). For each condition, the donor most representative of the group mean (closest IDM value) is shown.

**Figure 3:**
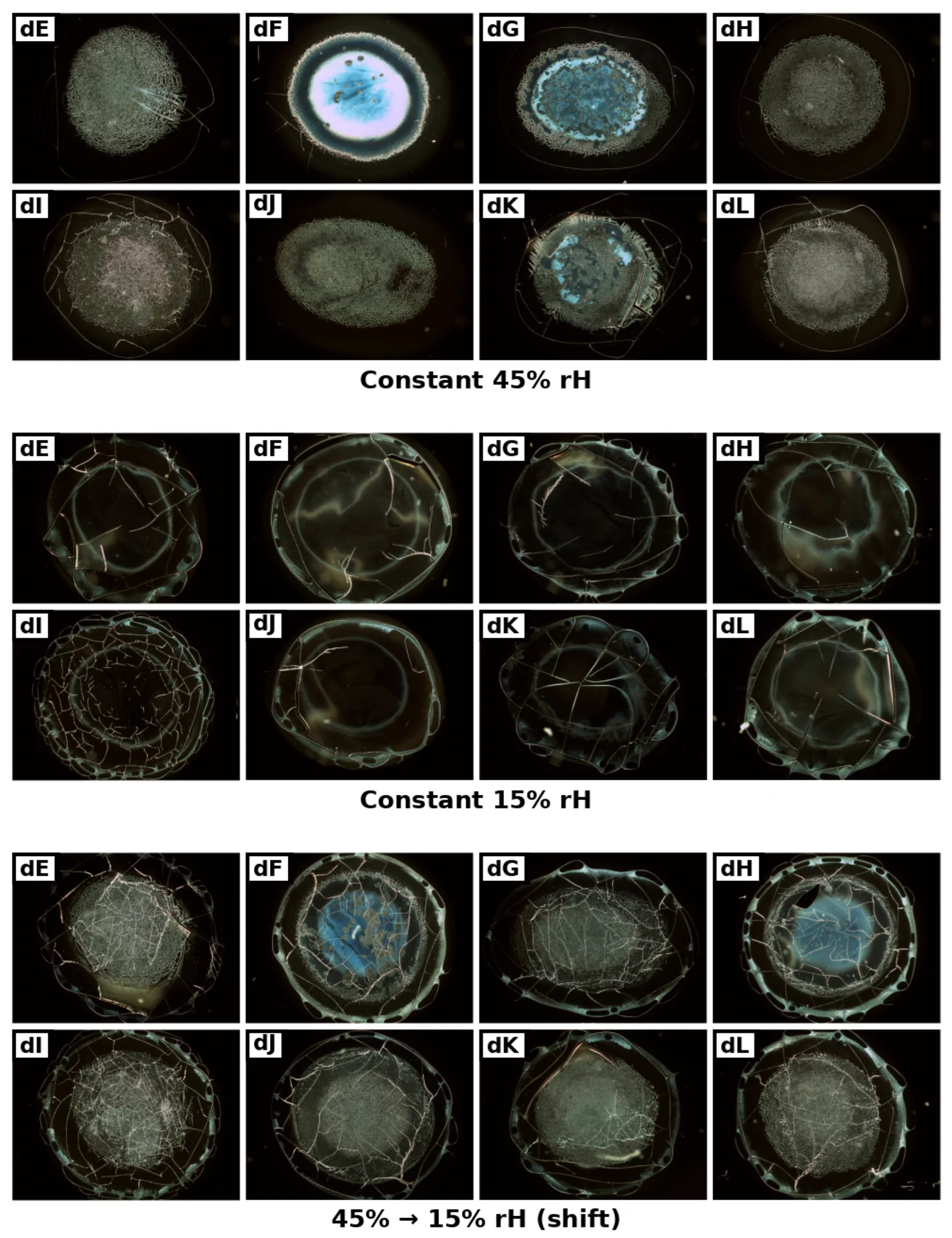
Representative dark-field images of dried plasma droplets from all eight donors (dE–dL), shown for each of the three evaporation conditions (**top** to **bottom**: constant 45% rH, constant 15% rH, and the 45% → 15% rH shift). For each condition, the donor most representative of the group mean (closest IDM value) is shown.

Across both fluids, two characteristic structural features were observed in the dried droplet residues: crystalline structures, located predominantly in the inner zone of the residue, and cracks, located predominantly at the periphery. The relative development of these two features depended strongly on the evaporation condition applied (Figure 1, Figure 2 and Figure 3).

Under constant 15% rH, the inner area of the droplet residues was largely structure-free or filled with a faint, structureless haze, while the periphery contained numerous cracks of variable thickness (Figure 1a).

Under constant 45% rH, the droplet residues developed well-formed crystalline structures occupying the central area, arranged in island-like clusters separated by structure-free regions, with no or only a few, fine, often circularly arranged cracks at the periphery (Figure 1c).

Under the rH-shift condition (45% → 15%), residues of both fluid droplets combined both characteristic features, with well-developed crystalline structures in the inner zone and well-developed cracks at the periphery, present together within the same droplet residue. On visual inspection, these patterns showed a greater variability between donors than those formed under either stable condition (Figure 1b). 

Figure 2 shows representative images of dried serum droplets from all eight donors, illustrating the donor-to-donor variability under each evaporation condition and showing the greatest visual diversity between the donors apparent under the rH-shift condition. Figure 3 shows the corresponding images for plasma, which likewise illustrate the group-wide consistency of the condition-dependent pattern differences. For each condition in Figure 2 and Figure 3, the pattern shown is the one whose IDM (inverse difference moment) value was closest to the group mean for that condition; IDM was chosen for this purpose as it best reflects the overall homogeneity, and therefore typicality, of a given pattern among the texture parameters measured.

### 2.2. Deep Learning-Based Donor Classification

The CNN classification results should be interpreted as an exploratory assessment of the feasibility of using deep learning to discriminate donor-specific droplet patterns, rather than as the development of a clinically applicable donor identification system.

Table 1 summarizes the CNN-based donor classification results for serum and plasma droplet patterns obtained under the three evaporation conditions.

For serum, the donor classification accuracy was 80.8% under stable 45% rH, 66.2% under stable 15% rH, and 84.4% under the rH-shift condition. For plasma, the accuracy was 67.5% under stable 45% rH, 70.5% under stable 15% rH, and 83.3% under the rH-shift condition. In both fluids, therefore, the highest classification accuracy, and thus the highest amount of donor-specific information recoverable by the CNN, was obtained under the rH-shift condition, while the lowest accuracy was obtained under one of the two stable conditions (15% rH for serum, 45% rH for plasma).

**Figure 4:**
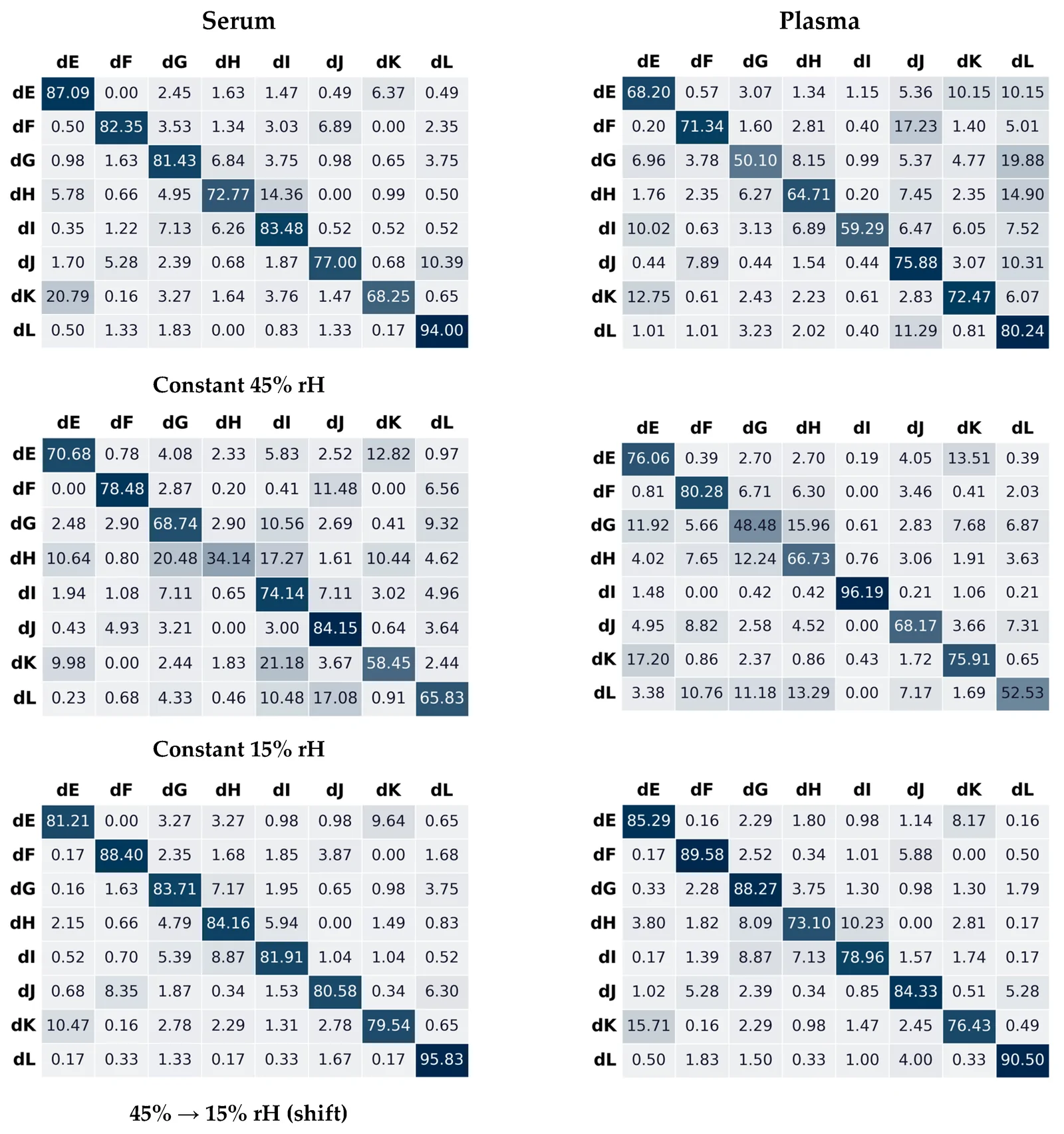
Confusion matrices (% of test patches) for the eight-class donor classification (donors dE–pL) by the convolutional neural network, for serum and plasma under each of the three evaporation conditions. Diagonal values (darkest cells) indicate the percentage of patches correctly assigned to the true donor; off-diagonal values indicate the percentage misclassified as each other donor. Cell shading scales with this same percentage, from light (**low**) to dark (**high**).

Inspection of the confusion matrices (Figure 4) showed that misclassifications were not evenly distributed across donor pairs but tended to concentrate on a small number of donor pairs per condition, and that these pairs differed between conditions. For both fluids, the highest number of such misclassifications across donor pairs was observed for the stable 15% rH condition (10 and 8 pairs with more than 10% misclassifications for serum and plasma, respectively), followed by the stable 45% rH condition (3 and 9), whereas the rH-shift condition showed the lowest number of such pairs (1 and 2). These observations indicate that the rH-shift condition did not merely increase the average classification accuracy but specifically improved the separability of donor pairs that were difficult to distinguish under stable conditions.

To assess whether classification performance differed among evaporation conditions at the donor level, classification accuracies for each of the eight donors (i.e., the diagonal values of the confusion matrices in Figure 4) were compared using Friedman tests. Significant differences among conditions were observed for both serum (χ^2^(2) = 7.75, *p* = 0.021) and plasma (χ^2^(2) = 9.75, *p* = 0.008). Post hoc paired Wilcoxon signed-rank tests with Holm correction showed that, for serum, accuracy at stable 15% rH was significantly lower than at stable 45% rH (adjusted *p* = 0.047) and under the rH-shift condition (adjusted *p* = 0.047), whereas stable 45% rH and the rH-shift condition did not differ significantly (adjusted *p* = 0.109). For plasma, the rH-shift condition showed significantly higher accuracy than stable 45% rH (adjusted *p* = 0.023); the remaining pairwise comparisons were not significant after correction (adjusted *p* ≥ 0.219).

The above results indicate that donor classification by the CNN was sensitive to the evaporation environment, while the rH-shift condition consistently yielded the highest donor-level accuracy in both serum and plasma. Thus, for the exploratory purposes of this study, the CNN results support the presence of donor-specific morphological information in the dried-droplet patterns and suggest that sequential humidity exposure may enhance its recoverability.

Overall, these findings provide exploratory evidence that CNNs can successfully exploit donor-dependent morphological information contained in the droplet residues, with classification performance differing significantly among evaporation conditions for both fluids, and the rH-shift condition yielding the highest overall accuracy. Future studies including larger and more diverse populations will be required to determine the robustness and generalizability of this approach.

### 2.3. Correlations Between Image-Evaluation Parameters and Blood Parameters

The image-evaluation parameters obtained from the main experiments correlated with several serum and plasma characteristics (Table 2). Across both fluids and all evaporation conditions, 179 correlations with |r| = 0.55–1 were identified; these are hereafter referred to as strong correlations. Among them, 25 were associated with triglycerides, 24 with total protein, 21 with age, and 20 with urea. The strongest correlations (|r| = 0.85–1) were observed for triglycerides (7 of 21), total protein (4 of 21), and urea (3 of 21). Among the pattern-evaluation parameters, lacunarity (22/179), local connected fractal dimension (21/179), and inverse difference moment (20/179) exhibited the highest numbers of strong correlations. The highest numbers of the strongest correlations were observed for the box-count fractal dimension (5/21), gray-level distribution (3/21), and local connected fractal dimension (3/21).

In general, more strong correlations between the pattern-evaluation parameters and the corresponding serum or plasma characteristics were found for plasma (102) than for serum (77). The highest number of strong correlations was observed for plasma under the 15% rH condition (40/102), whereas the lowest number was observed for serum under the same condition (22/77). This finding suggests that low humidity amplified the signal in plasma but dampened it in serum. The opposite responses of the two fluids may be related to the presence of fibrinogen in plasma, which is absent in serum. Under the 45% rH condition, 35 of 102 strong correlations were observed for plasma and 28 of 77 for serum. Under the rH-shift condition, the number of strong correlations was identical for both fluids, amounting to 27 correlations in serum (27/77) and 27 in plasma (27/102).

Among the 179 strong correlations, negative correlations (96) were more frequent than positive correlations (83). Among the pattern-evaluation parameters, the local connected fractal dimension exhibited the highest number of negative correlations (13), whereas entropy showed the highest number of positive correlations (10). Among the fluid characteristics, triglycerides were associated with the highest number of positive correlations (16), while age, urea, and glucose exhibited the highest numbers of negative correlations (13, 12, and 11, respectively).

Across all evaporation conditions, 11 strong correlations changed sign. Seven of these sign reversals occurred between the two stable humidity conditions, whereas four occurred between the 45% rH and rH-shift conditions. In serum, four of the five sign reversals occurred between the 45% rH and rH-shift conditions. In contrast, all six sign reversals observed in plasma occurred between the two stable humidity conditions.

Given the small number of donors (*n* = 8), these correlations should be regarded as exploratory and hypothesis-generating rather than confirmatory. They are reported here to illustrate the potential physiological relevance of DEM image parameters and to motivate future studies with larger sample sizes.

## 3. Discussion

The present results show that patterns formed by dried serum and plasma droplets have a higher donor-discrimination potential when a relative-humidity shift is applied during the evaporation phase (45% rH for 2 h, followed by a shift to 15% rH until complete drying) than patterns obtained under constant conditions (45% or 15% rH throughout the entire drying process). Under constant conditions, the two characteristic features of serum and plasma patterns, i.e., crystalline structures located in the center of the droplet and cracks, reach their full development at high and low rH, respectively. In contrast, droplets dried under changing humidity conditions exhibit both well-developed structural features simultaneously. This observation is consistent with the previously described dynamics of evaporating biological-fluid droplets, including the segregation process, in which small molecules accumulate predominantly in the droplet center [17], and the occurrence of evaporation in distinct stages [18,19,20].

The segregation process occurs during the initial stage of evaporation, characterized by the constant-rate evaporation of free, mobile water, while the droplet is still in a liquid state. During this stage, small molecules, such as salts, are transported toward the droplet center and, in subsequent evaporation stages, contribute to the formation of dendritic crystalline structures. Larger molecules, such as proteins, in contrast, become more evenly distributed throughout the droplet. Once the evaporation of free water is complete, the droplet enters the gelation stage, during which crystalline structures begin to form. The final, falling-rate evaporation stage is associated with the evaporation of bound water. During this stage, mechanical stresses develop within the protein film, leading to crack formation.

The rH-shift protocol used here can therefore be understood as modeling these successive evaporation stages within the droplets. The initial 2 h high-rH phase corresponds to the free-water-dominated evaporation and gelation stages, allowing the segregation process and the resulting crystalline structures to develop fully before the subsequent low-rH phase promotes bound-water evaporation and the associated mechanical stresses. This, in turn, facilitates the formation of well-developed peripheral cracks in addition to the already formed crystalline structures. Because the timing of the transition from free- to bound-water evaporation depends on droplet volume, the switching time in the rH-shift protocol would need to be adjusted accordingly for droplets of different sizes.

The CNN-based classification results indicate that this increase in pattern complexity under the rH-shift condition is accompanied by an increase in the amount of donor-specific information that can be recovered from the images for both serum and plasma. The rH-shift condition yielded the highest classification accuracy among the three evaporation conditions in both fluids (84.4% for serum and 83.3% for plasma), exceeding the best stable condition by 3.6 percentage points for serum (80.8% at 45% rH) and by 12.8 percentage points for plasma (70.5% at 15% rH). Analysis of the confusion matrices further suggests that the rH shift improved the separability of donor pairs that were poorly distinguished under stable conditions, rather than producing a uniform improvement across all donors. A plausible explanation is that the combination of two structurally distinct and well-developed features within a single pattern provides the CNN with two at least partially independent sources of donor-specific information: the inner crystalline region and the peripheral crack region. Each of these regions may be particularly informative for different donor pairs.

The correlations observed between DEM image-evaluation parameters and serum or plasma characteristics suggest that the composition of the respective fluid is at least partly reflected in the resulting dried-droplet patterns. Of the 840 image-parameter–fluid-parameter pairs examined, 179 (21%) reached at least moderate correlation strength (|r| = 0.55–1), with triglycerides, total protein, age, and urea most frequently involved, and fractal-geometry parameters (lacunarity, local connected fractal dimension, and mass and box-count fractal dimensions) contributing the largest share of these associations. Plasma yielded more strong correlations than serum overall (102 vs. 77), and this difference was condition-dependent: under 15% rH, plasma showed the highest number of strong correlations of any condition (40) while serum showed the lowest (22), whereas under the rH-shift condition the two fluids were balanced (27 each). A plausible explanation for this fluid-specific response to humidity is the presence of fibrinogen in plasma, which is absent in serum and may influence how the protein network develops under different drying regimes. Sign reversals between conditions were comparatively rare (11 of 179 strong correlations) and followed distinct patterns in the two fluids: in serum, reversals occurred predominantly when the rH-shift condition was compared with either stable condition, whereas in plasma they occurred predominantly between the two stable conditions themselves, primarily involving lacunarity.

It is worth clarifying how the donor-classification task should be understood. It is not meant as an identity-verification application. Instead, classification accuracy is used here as a proxy for how much donor-specific information the dried droplet pattern contains, since pattern formation depends, among other things, on the biochemical and physicochemical makeup of the drying fluid. Under this view, the higher classification accuracy seen under the rH-shift condition means that the pattern carries more information overall—an important step toward diagnostic usefulness, but not enough on its own to prove it. This relationship is not necessarily straightforward, however. By making the pattern more sensitive to differences between donors in general, the rH-shift condition may also add variability that has nothing to do with any particular disease. This could work against disease detection in two ways: a disease signal that is small compared with this added variability could simply get diluted, or the disease signal may depend on different pattern features than the ones the rH-shift condition amplifies, in which case a different evaporation regime could pick it up better. The correlation results above support this idea: although the rH-shift condition gave the best donor discrimination, it did not produce the highest number of strong correlations with blood-derived physiological parameters, and the specific parameters it did correlate with often differed from those seen under stable conditions—for serum, for example, both constant conditions correlated strongly with total protein, while the rH-shift condition instead correlated strongly with homocysteine. A constant-humidity condition, which captures fewer dimensions of donor variation overall, might therefore give a better signal-to-noise ratio for diseases related to protein content in blood, such as those marked by altered total protein levels. Which evaporation condition works best is therefore likely to depend on the specific disease, and this should be tested directly in future studies involving donors with known clinical conditions, rather than assumed from donor-classification accuracy alone.

This study has several limitations. First, the sample comprised only eight donors, predominantly women (six of eight), all of whom were apparently healthy. For this reason, the correlational findings should be regarded as exploratory and hypothesis-generating rather than confirmatory. The small and relatively homogeneous donor cohort limits the generalizability of the findings. Accordingly, the CNN classification results should be interpreted as a proof-of-concept demonstration that donor-specific morphological information can be recovered from the droplet patterns, rather than as an estimate of diagnostic performance in a broader population. The CNN was also trained and tested separately for each fluid and evaporation condition using datasets derived from a limited number of experimental days. Larger and more diverse cohorts, together with independent replication, will be required to determine whether the observed effects of evaporation conditions on donor classification are generalizable.

Taken together, the present findings suggest that deliberately varying rH during droplet evaporation, rather than maintaining it at a constant level as in previous DEM studies, can increase both the visual complexity of the resulting patterns and the amount of donor-specific information that a CNN can extract from them. This raises the possibility that rH-shift protocols, evaluated using deep learning, could improve the sensitivity of DEM-based diagnostic pattern tests. Transitioning from donor identification to disease discrimination would require models trained to recognize disease-related patterns that generalize across individuals, supported by larger clinically labeled datasets. Transfer learning could support this transition by fine-tuning representations learned from DEM images for disease classification, while domain adaptation could help account for differences across populations, imaging systems, and experimental conditions. Future studies should test this hypothesis in larger and more diverse cohorts, including donors with clinically relevant diseases.

## 4. Materials and Methods

### 4.1. Ethics and Sample Collection

The study was conducted within the scope of the Swiss Human Research Act (Section 2, § 1) and was approved by the Ethics Commission of Nord-West and Central Switzerland (Project ID 2022-02043) on 14 December 2022. Blood was collected from eight apparently healthy donors (six women, two men) referred to individually as donors dE–dL, who had given written informed consent. Blood collection was performed by a trained study nurse at the Society for Cancer Research (Arlesheim, Switzerland). Samples were blinded by an independent person using a letter code.

From each donor, venous blood was collected into a serum separator tube with clot activator and into a K2-EDTA tube. The serum tube was centrifuged for 10 min at 2000× *g*, and the EDTA tube for 15 min at 2000× *g*. Serum and plasma were divided into 0.2 mL portions and stored at −21 °C until analysis.

### 4.2. Droplet Evaporation Method

For droplet evaporation, microscope slides (76 × 26 mm, pre-cleaned, cut edges) were cleaned in four consecutive baths (one bath of 75% ethanol, three baths of purified water, ≥5 min each) and dried with a lint-free laboratory wiper. Fourteen droplets of 2 μL each were deposited on each slide, in two rows of seven, using a micropipette. Droplet evaporation was carried out in a climate exposure test cabinet with controlled temperature and humidity (KBF 240, WTB Binder Labortechnik, Tuttlingen, Germany), which contained two inner plexiglass chambers (upper and lower) covered with semi-permeable foam and placed on a vibration-damping support. In each experiment, 24 slides (16 in the upper chamber, 8 in the lower chamber, placed in rows of four slides each) were arranged following a quasi-randomization design in order to minimize the possible effects of spatial gradients within the chambers.

### 4.3. Evaporation Conditions

Three evaporation conditions were applied to serum and to plasma, each replicated on three independent experimentation days (18 main experiments in total): (1) constant 45% rH for 2 h; (2) constant 15% rH for 2 h; and (3) an rH shift from 45% to 15%, comprising 2 h at 45% rH followed by evaporation at 15% rH until completely dry. Temperature was held constant within each fluid type, at 24.5 °C for serum and 30.5 °C for plasma, corresponding to the optimal temperatures identified in a previous study for these two fluids [2].

### 4.4. Image Acquisition

After evaporation, droplet residues were inspected with an optical microscope in dark-field mode (Zeiss Lab.A1, Carl Zeiss Microscopy, Jena, Germany) at 25× magnification and photographed with an attached camera (Moticam 5.0 MP, Motic Electric Group, Xiamen, China). Images were saved as JPG files (1360 × 1024 pixels). Images showing major artifacts (e.g., contamination) were excluded from further analysis. The experiments yielded a total of 1001, 996, and 1000 patterns for the evaporation conditions 45% rH (constant), 15% rH (constant), and shift from 45% to 15% rH, respectively.

### 4.5. Computerized Texture and Fractal Image Analysis

Whole images (1360 × 1024 pixels) of the droplet residues were used for analysis. Images were converted from RGB to 8-bit grayscale using ImageJ’s default (unweighted) conversion. Texture parameters (grey-level distribution, contrast, correlation, inverse difference moment, and entropy) were obtained from these 8-bit images using the GLCM Texture plugin (pixel-pair distance of four pixels, angle of 90°). For fractal analysis, images were binarized using ImageJ’s built-in “Default” automatic thresholding algorithm, applied identically to all images, and analyzed with the FracLac plugin (odd-size scaling, box sizes of 4–40 pixels) to obtain the number of foreground pixels, local connected fractal dimension (LCFD), mass fractal dimension (MFD), box-counting fractal dimension (BCFD), and lacunarity. All image processing steps were applied in ImageJ (v.1.50b). The resulting parameters were correlated with the serum and plasma blood-analysis results (Section 4.7 and Section 4.8).

### 4.6. Patch Extraction and CNN Training for Donor Classification

To explore whether the morphological differences induced by the different evaporation conditions contained donor-specific information, a DenseNet121 convolutional neural network (CNN) was employed [21,22,23]. The CNN was used as an analytical tool to quantify the donor differentiation potential of the generated droplet patterns under the different evaporation conditions. For each combination of fluid (serum or plasma) and evaporation condition (constant 45% rH, constant 15% rH, and sequential 45% → 15% rH), the corresponding droplet residue images were used for donor classification using the CNN.

To capture local morphological features while matching the network input size, each original image (1360 × 1024 pixels) was divided into multiple 256 × 256-pixel full-content patches (i.e., patches with background content were discarded). Partially overlapping windows were applied to improve the spatial coverage of the droplet morphology. Beyond adapting the images to the CNN input size, patch extraction was adopted as a data expansion strategy. Because the dataset comprised approximately 125 droplet images per donor, the number of original samples available for training an eight-class classifier was relatively modest. Generating multiple patches from each image effectively increased the number of training examples and enhanced the network’s ability to learn discriminative local morphological features. The original images were randomly partitioned into training (80%) and test (20%) subsets before patch extraction, ensuring that all patches originating from the same image remained within the same subset. Within the training set, 10% of the data was used for validation during model training. No conventional data augmentation or cross-validation was applied.

The DenseNet121 model was configured as an eight-class classifier, with each class representing one donor (dE–dL). The model was optimized using the Adam optimizer with categorical cross-entropy as the loss function, and early stopping based on validation performance was applied to prevent overfitting. Model performance was evaluated using overall test accuracy, while confusion matrices were used to visualize the distribution of predictions across donors. The resulting classification performance was interpreted as a quantitative measure of the donor-specific information encoded in the droplet patterns generated under each evaporation condition.

### 4.7. Serum and Plasma Analysis

Serum and plasma samples from the eight donors used in the main experiments were sent to SwissAnalysis AG (Tägerwilen, Switzerland) for the determination of potassium, creatinine, urea, total bilirubin, glomerular filtration rate (GFR), glucose, total protein, triglycerides, homocysteine, glycated hemoglobin (HbA1c), high-sensitivity C-reactive protein (CRP), calcium, and phosphate. Results were anonymized and coded in the same way as the DEM samples.

### 4.8. Statistical Analysis

Correlations between image-evaluation outcomes and blood parameters were assessed by means of the Pearson linear correlation coefficient r. Correlation coefficients of r = 0.55 to 1 (positive) or r = −1 to −0.55 (negative) were considered to represent correlations of at least moderate strength. Because of the small sample size (*n* = 8 donors), these correlations should be regarded as exploratory and hypothesis-generating rather than confirmatory.

For the CNN-based donor classification, the independent biological sample size was defined by the eight donors rather than by the number of images or patches. Classification accuracies for each donor were therefore compared across the three evaporation conditions using Friedman tests, followed by paired Wilcoxon signed-rank tests with the Holm correction for multiple comparisons. Given the small donor cohort and the exploratory design of the study, no a priori power calculation was performed, and the CNN classification results should be interpreted as a proof-of-concept assessment of donor-specific information rather than as a generalizable estimate of diagnostic performance.

## 5. Conclusions

In serum and plasma droplets dried under a shift from 45% to 15% relative humidity, the two structural features characteristic of dried residues of blood-derived fluids—inner crystalline structures and peripheral cracks—developed simultaneously and in a more elaborate form than under either constant relative humidity condition. A convolutional neural network trained to recognize donor identity from these patterns achieved its highest classification accuracy under the rH-shift condition for both fluids (84.4% for serum and 83.3% for plasma) and specifically improved the separability of donor pairs that were poorly distinguished under stable conditions. Image-evaluation parameters further correlated with several serum and plasma characteristics, providing preliminary evidence for a link between DEM patterns and donor physiology. These findings support rH-shift DEM, combined with deep learning-based pattern evaluation, as a promising direction for the development of diagnostic pattern tests, although confirmation in larger cohorts is required.

## Figures and Tables

**Table 1:** Convolutional neural network classification accuracy for donor identity (eight donors, E−L), by fluid and evaporation condition.is a table.

Fluid	Condition	Test Patches (*n*)	Correctly Classified (*n*)	Accuracy (%)
Serum	45% rH (stable)	3968	3204	80.8%
Serum	15% rH (stable)	3872	2562	66.2%
Serum	45% → 15% rH (shift)	4800	4052	84.4%
Plasma	45% rH (stable)	3968	2680	67.5%
Plasma	15% rH (stable)	3904	2751	70.5%
Plasma	45% → 15% rH (shift)	4800	3999	83.3%

**Table 2:** Correlations between donor data (age, blood analysis results) and computerized image evaluation parameters for serum and plasma under the three humidity conditions. Correlations with |r| ≥ 0.55 are shaded; the shading color is green for positive and purple for negative correlations, with three color intensities reflecting correlation strength: |r| = 0.55–0.69 (light), 0.70–0.84 (medium), 0.85–1 (intense). Abbreviations: GLD, gray-level distribution; IDM, inverse difference moment; Nr fg pixel, number of foreground pixels; LCFD, local connected fractal dimension; MFD, mass fractal dimension; BCFD, box-counting fractal dimension; GFR, glomerular filtration rate; HbA1c, glycated hemoglobin; CRP ultrasensitive, high-sensitivity C-reactive protein.

	Age Years	Potassium mmol/L	Creatinine µmol/L	Urea mmol/L	Tot Bilirubin µmol/L	GFR mL/min	Glucose mmol/L	Total Protein g/L	Triglycerides mmol/L	Homocysteine µmol/L	HbA1c %	CRP Ultrasensitive mg/L	Calcium in Serum mmol/L	Phosphate mmol/L
Serum: constant 45% rH														
GLD	−0.83	−0.45	0.61	−0.64	0.1	−0.17	−0.58	0.51	−0.08	0.61	−0.46	−0.37	0.14	0.12
Contrast	−0.08	−0.15	0.13	−0.25	−0.12	−0.3	−0.85	−0.1	0.08	0.49	−0.61	−0.3	0	−0.68
Correlation	−0.12	−0.41	−0.17	−0.06	0.19	0.34	−0.71	−0.16	0.33	−0.06	−0.54	−0.3	0.47	−0.57
IDM	−0.07	0.3	0.29	−0.17	0.52	−0.22	0.14	0.4	−0.79	0.21	0.49	−0.08	−0.1	−0.09
Entropy	0.21	−0.09	0	0.36	−0.27	−0.32	−0.13	−0.39	0.67	0.14	−0.31	0.03	0	0.12
Nr fg pixel	−0.75	0.16	0.51	−0.58	0.23	−0.09	0	0.96	−0.5	0.58	0.24	−0.16	0.31	0.22
LCFD	−0.32	0.32	0.34	−0.21	0.18	−0.14	0.52	0.6	−0.67	0.21	0.64	−0.07	−0.05	0.38
MFD	0.56	−0.08	−0.44	0.68	−0.23	0.11	0.07	−0.69	0.78	−0.42	−0.14	0.12	0.16	−0.02
BCFD	0.26	0.5	0.41	0.39	0.22	−0.9	0.38	0	−0.35	0.5	0.59	−0.19	−0.29	0.17
Lacunarity	0.36	−0.21	−0.28	0.27	−0.08	−0.06	−0.56	−0.64	0.49	−0.05	−0.54	−0.2	0.02	−0.6
Serum: constant 15% rH														
GLD	−0.25	−0.28	0.11	0.02	−0.31	0.1	0.16	0.06	0.51	−0.07	−0.18	0.2	0.12	0.65
Contrast	−0.01	−0.01	0.3	−0.06	−0.1	−0.07	0.37	0.39	0.23	0.05	0.11	0.56	0.02	0.89
Correlation	−0.5	−0.61	−0.17	−0.53	0.08	0.8	−0.49	0.11	−0.12	−0.35	−0.45	−0.28	0.27	−0.34
IDM	−0.08	0.33	0.26	−0.24	−0.48	−0.21	0.02	0.44	−0.71	0.28	0.37	−0.01	−0.06	−0.18
Entropy	0.09	−0.28	−0.18	0.27	−0.44	0.14	0.07	−0.37	0.71	−0.25	−0.3	0.12	0.05	0.34
Nr fg pixel	−0.51	0.34	0.44	−0.22	0.34	−0.12	0.33	0.91	−0.4	0.43	0.56	0.01	0.44	0.43
LCFD	−0.6	0.02	0.45	−0.33	0.63	−0.03	−0.13	0.7	−0.67	0.4	0.37	−0.53	0.45	−0.11
MFD	0.54	−0.15	−0.53	0.29	−0.59	0.1	−0.19	−0.82	0.66	−0.35	−0.55	0.16	−0.33	−0.3
BCFD	0.05	−0.01	0.5	0.4	0.52	−0.51	0.54	−0.03	−0.52	0.02	0.72	−0.33	−0.21	0.54
Lacunarity	0.6	−0.07	−0.51	0.35	−0.57	0	−0.11	−0.85	0.6	−0.31	−0.45	0.12	−0.38	−0.3
Serum: 45% → 15% rH (shift)														
GLD	−0.85	−0.42	0.64	−0.63	0.09	−0.17	−0.45	0.57	−0.09	0.59	−0.38	−0.28	0.12	0.28
Contrast	−0.27	0	0.36	−0.33	0.21	−0.48	−0.76	0.23	−0.05	0.77	−0.42	−0.33	0.11	−0.51
Correlation	−0.33	−0.6	−0.26	−0.25	−0.1	0.65	−0.6	−0.11	0.42	−0.26	−0.65	−0.24	0.44	−0.34
IDM	0.39	0.56	−0.11	0.23	0.31	−0.16	0.34	0.08	−0.44	−0.02	0.58	0.13	−0.04	−0.21
Entropy	−0.61	−0.54	0.41	−0.4	−0.06	−0.08	−0.52	0.19	0.23	0.37	−0.56	−0.27	0.11	0.17
Nr fg pixel	0.6	0.15	−0.59	0.66	−0.32	0.32	0.37	−0.47	0.79	−0.57	0.05	0.51	0.25	0.18
LCFD	0.28	−0.23	−0.41	0.35	−0.14	0.6	0.5	−0.34	0.31	−0.86	0.15	0.42	0.03	0.47
MFD	−0.32	0.06	0.37	−0.47	−0.04	−0.44	−0.31	0.21	−0.51	0.6	−0.13	−0.44	−0.38	−0.31
BCFD	0.33	−0.19	−0.5	0.32	0	0.69	0.36	−0.33	0.22	−0.89	0.13	0.39	0.12	0.2
Lacunarity	−0.26	0.17	0.34	−0.38	0.13	−0.5	−0.53	0.26	−0.38	0.76	−0.18	−0.47	−0.09	−0.57
Plasma: constant 45% rH														
GLD	0.31	−0.29	−0.4	0.47	−0.11	0.28	−0.23	−0.57	0.72	−0.38	−0.33	−0.06	0.34	−0.21
Contrast	0.2	0.33	0.31	0.43	0.29	−0.78	0.07	−0.13	−0.26	0.51	0.42	−0.6	−0.03	−0.22
Correlation	−0.39	−0.24	0.01	−0.29	0.21	0.24	−0.75	0.24	0.16	0.28	−0.45	−0.38	0.61	−0.58
IDM	−0.63	−0.05	0.71	−0.65	0.57	−0.3	−0.23	0.75	−0.82	0.55	0.14	−0.18	−0.08	0.11
Entropy	0.5	0.03	−0.62	0.64	−0.49	0.24	0.12	−0.69	0.78	−0.4	−0.13	−0.1	0.23	−0.23
Nr fg pixel	−0.01	0.76	0.18	−0.07	−0.15	−0.44	0.47	0.54	−0.28	0.52	0.51	0.24	−0.07	0.21
LCFD	0.64	−0.2	−0.72	0.32	−0.53	0.47	0.04	−0.85	0.6	−0.78	−0.41	0.44	−0.33	−0.16
MFD	−0.2	−0.34	0.06	0.36	0.32	0.34	0.29	0.04	0.16	−0.38	0.32	−0.24	0.57	0.46
BCFD	−0.1	−0.43	0.01	−0.62	−0.31	0.03	−0.65	−0.24	0	0.05	−0.86	0.18	−0.66	−0.29
Lacunarity	−0.67	−0.04	0.83	−0.57	0.53	−0.54	−0.3	0.71	−0.74	0.79	0.11	−0.41	−0.06	0.07
Plasma: constant 15% rH														
GLD	0.49	−0.06	−0.49	0.48	−0.32	0.17	−0.16	−0.58	0.87	−0.3	−0.39	0.26	0.2	−0.18
Contrast	0.35	0.36	−0.33	0.8	−0.05	0.16	0.73	−0.1	0.43	−0.35	0.65	0.07	0.56	0.36
Correlation	−0.45	−0.14	−0.08	−0.34	0.01	0.31	−0.67	0.32	0.25	0.31	−0.45	−0.36	0.67	−0.57
IDM	−0.58	−0.07	0.59	−0.61	0.5	−0.16	−0.05	0.66	−0.91	0.35	0.25	−0.21	−0.16	0.14
Entropy	0.56	0.08	−0.55	0.6	−0.42	0.14	0.02	−0.59	0.92	−0.3	−0.25	0.25	0.23	−0.13
Nr fg pixel	−0.63	−0.15	0.51	−0.4	0.3	0.1	0.33	0.72	−0.46	0.07	0.35	0.1	0.1	0.75
LCFD	−0.63	−0.51	0.48	−0.91	0.04	−0.02	−0.61	0.38	−0.28	0.31	−0.66	0.15	−0.39	0.12
MFD	0.68	0.13	−0.6	0.53	−0.29	0.03	−0.23	−0.74	0.63	−0.22	−0.31	0.02	0.05	−0.62
BCFD	−0.72	−0.52	0.57	−0.91	0.12	−0.01	−0.49	0.49	−0.39	0.31	−0.51	−0.11	−0.34	0.26
Lacunarity	0.63	0.16	−0.59	0.59	−0.24	0.06	−0.18	−0.66	0.66	−0.2	−0.21	−0.06	0.23	−0.6
Plasma: 45% → 15% rH (shift)														
GLD	0.33	−0.08	−0.37	0.3	−0.38	0.05	−0.34	−0.47	0.86	−0.06	−0.54	0.16	0.18	−0.25
Contrast	0.3	0.21	0.14	0.76	0.23	−0.54	0.18	−0.23	0.35	0.25	0.36	−0.32	0.36	0.09
Correlation	−0.14	0.22	−0.04	−0.07	0.06	−0.09	−0.56	0.26	0.32	0.53	−0.28	−0.21	0.61	−0.58
IDM	−0.58	−0.06	0.52	−0.67	0.46	−0.09	−0.18	0.66	−0.87	0.37	0.14	−0.21	−0.11	−0.02
Entropy	0.58	0.07	−0.53	0.58	−0.53	0.05	0.04	−0.67	0.88	−0.28	−0.28	0.21	0.05	−0.11
Nr fg pixel	−0.16	−0.05	0.08	−0.09	0.39	0.37	0.44	0.33	−0.5	−0.4	0.5	0.17	0.08	0.4
LCFD	−0.46	−0.48	−0.14	−0.78	−0.06	0.73	−0.47	0.24	−0.19	−0.29	−0.55	0.15	−0.02	−0.23
MFD	0.5	0.06	−0.6	0.4	−0.7	0.24	0.14	−0.56	0.91	−0.39	−0.34	0.47	−0.03	0.06
BCFD	−0.8	−0.55	0.68	−0.86	0.53	0.02	−0.48	0.6	−0.72	0.28	−0.24	−0.18	−0.15	0.15
Lacunarity	0.24	0.15	0.33	0.48	0.16	−0.8	−0.04	−0.26	0.17	0.48	0.12	−0.31	−0.05	0.03

## Data Availability

The data presented in this study are available from the corresponding author upon reasonable request.

## Abbreviations

The following abbreviations are used in this manuscript:

CNN	convolutional neural network
DEM	Droplet evaporation method
